# Effects of Different Irrigation Solutions and Protocols on Mineral Content and Ultrastructure of Root Canal Dentine

**DOI:** 10.22037/iej.v13i2.19287

**Published:** 2018

**Authors:** Brenna Magdalena Lima Nogueira, Thais Isabele da Costa Pereira, Victor Feliz Pedrinha, Patrícia de Almeida Rodrigues

**Affiliations:** a School of Dentistry, Federal University of Pará, Belém, Pará, Brazil

**Keywords:** Chelating Agent, Dentin, Root Canal Irrigant, Surface Properties

## Abstract

**Introduction:**

The aim of this study was to evaluate the effect of different irrigating solutions and irrigation protocols on the chemical and physical structure of root dentin.

**Materials and Methods:**

Thirty extracted single-rooted human teeth were selected and then distributed into the following treatment groups (*n*=10): G1, saline solution (0.9% NaCl); G2, 2.5% NaOCl + 17% EDTA + 2.5% NaOCl; G3, 2.5% NaOCl + 9% Etidronate (HEBP) + 2.5% NaOCl; G4, mixture of 5% NaOCl + 18% HEBP; G5, 2.5% NaOCl + 17% EDTA + 0.9% NaCl, and G6, 2.5% NaOCl + 9% HEBP + 0.9% NaCl. The ultrastructure of dentin was characterized through scanning electron microscopy (SEM) and energy dispersive x-ray spectrometry (EDS) determined the chemical composition in terms of the calcium (Ca), phosphorus (P), magnesium (Mg) and potassium (K) content and the Ca/P ratio; and the crystalline phase was analyzed by x-ray diffraction (XRD). A descriptive analysis was performed on the ultrastructure and the crystallography data of dentin. Data analysis included a chemical composition analysis of variance (one-way ANOVA) and a subsequent multiple comparison test (Tukey’s test).

**Results:**

Except for the control group, all groups showed morphological changes upon visualization with SEM. For EDS, G2 and G5 showed significant mineral loss and changes in the Ca/P ratio (*P*<0.05); the highest values of Ca and P were observed in G3, G4 and G6.

**Conclusion:**

All the irrigating solutions and irrigation protocols tested promoted changes in the morphology and physical and chemical composition of the dentin. However, no significant differences were observed crystallographically.

## Introduction

Irrigating solutions are essential for cleaning and disinfecting root canals. The removal of necrotic tissue, microorganisms, bacterial products and the smear layer created during instrumentation is a major goal in the success of endodontic therapy [1, 2]. The use of sodium hypochlorite (NaOCl) at different concentrations and ethylenediaminetetraacetic acid (EDTA) have been recommended during chemomechanical preparation [3]. 

In this context, it was observed that when in contact with these substances, the dentin changes its physical, chemical and structural properties. NaOCl decreases the dentin microhardness [4], changes its flexural strength [5] and modulus of elasticity [5, 6], causes irreversible erosion of the dentin microstructure [7, 8] and oxidizes the organic matrix denaturing the collagen components of the dentin surface [9, 10]. Relating these facts to possibility of clinical occurrences, degradation of collagen matrix in mineralized tissues results in a less resistant and more brittle substrate, which can make the endodontically treated teeth more susceptible to crown or root fracture [11]. EDTA may negatively alter the microstructure of dentin. It can also change the original ratio of organic and inorganic components, resulting in undesirable changes in the properties of hardness and roughness, as well as causing dentin erosion [12, 13]. EDTA demineralizes the inorganic components of dentin by the chelation of calcium ions present in the hydroxyapatite, the main inorganic compound of dentin [4]. 

Etidronate (1-hydroxyethylidene-1, 1-bisphosphonate; HEBP) is a biocompatible chelating agent that can be used in combination with NaOCl without losing the desired properties of either compound [13, 14]. It interferes minimally with the physical properties of the dentin, such as microhardness [15] and roughness [16], and it prevents the formation of the smear layer during instrumentation [4, 8]. Despite the existence of results in the literature about microhardness and roughness properties [13, 15]. To the best of our knowledge, there are no studies about the effects of the mixture of HEBP and NaOCl on the organic and inorganic components of root dentin.

Dentin is composed of both organic and inorganic components. Any change in the ratio of calcium can significantly alter the original proportion of these components that influence the characteristics of dentin [17, 18]. Thus, the aim of this study was to evaluate the effect of different irrigating solutions (2.5% NaOCl, 17% EDTA and 9% HEBP) and irrigation protocols on the root dentin structure. The ultrastructure of dentin was characterized using scanning electron microscopy (SEM); the chemical composition was determined by energy dispersive x-ray spectrometry (EDS) analysis in terms of the calcium (Ca), phosphorus (P), magnesium (Mg), and potassium (K) content and the Ca/P ratio; and the crystalline phase was analyzed by x-ray diffraction (XRD). The null hypothesis was that there is no variation in the morphology, chemical composition and crystals of dentin after contact with different solutions and variable irrigation protocols.

## Materials and Methods

The substances evaluated in the present study were saline solution (sodium chloride 0.9%, NaCl), NaOCl, HEBP and EDTA. As a control, 0.9% NaCl was acquired from a pharmacy.

A stock solution of NaOCl (Sigma-Aldrich, St. Louis, MO, USA) was iodometrically titrated to determine its content of available chlorine. Then, the solution was diluted to 2.5% and 5% NaOCl, using distilled water. To obtain solutions of 9% and 18% HEBP (Zschimmer & Schwarz Mohsdorf GmbH & Co KG, Burgstadt, Germany), the pure chemical was mixed with deionized water. Concentrations of 5% and 18% were used to prepare a 1:1 mixture of NaOCl and HEBP, respectively, prior to experiments. The 17% EDTA (Sigma-Aldrich, St Louis, MO, USA) was prepared by dissolving disodium EDTA (Sigma-Aldrich, St Louis, MO, USA) in distilled water with the aid of sodium hydroxide (NaOH) (Sigma-Aldrich, St Louis, MO, USA); the pH was adjusted to 7.0 by adding hydrochloric acid (HCl) (Sigma-Aldrich, St Louis, MO, USA). All chemical substances were prepared and used just after the mixing.

All solutions were stored in dark containers at 5^° ^C between experiments. Before use, the substances were removed from the refrigerator and were allowed to equilibrate to room temperature for 60 min. 

**Table 1:** Division of groups according to irrigant solutions and irrigation protocols (*n*=10)

**Group**	**Initial irrigation (30 min)**	**Intermediate irrigation (30 min)**	**Final irrigation (1 min)**
**1**	Saline solution		
**2**	2.5% NaOCl	17% EDTA	2.5% NaOCl
**3**	2.5% NaOCl	9% HEBP	2.5% NaOCl
**4**	1:1 mixture of 5% NaOCl+18% HEBP (30 min)		
**5**	2.5% NaOCl	17% EDTA (3 min	2% CHX
**6**	2.5% NaOCl	9% HEBP (5 min)	2% CHX

**Table 2:** Comparison analysis by one-way ANOVA (*P*<0.0001) and multiple comparison analysis by the Tukey’s test (*P*<0.05) of the means and standard deviations of the chemical elements detected for the cervical third of the teeth in the different experimental groups (*n*=5)

**Groups**	**Chemical Elements of Root Dentin (w%)**				
	**K**	**Mg**	**Ca**	**P**	**Ca/P**
**G1**	0.031±0.20^A^	0.955±0.12^A^	35.912±1.08^A^	19.163±0.60^A^	1.741±0.02^A^
**G2**	0.028±0.02^A^	0.73±0.08^BD^	19.812±2.38^B^	10.483±1.41^B^	1.569±0.04^B^
**G3**	0.045±0.03^A^	0.827±0.05^AB^	29.536±1.18^CD^	18.123±0.53^A^	1.595±0.05^B^
**G4**	0.013±0.01^A^	1.203±0.06^C^	32.373±1.14^CE^	19.283±0.47^A^	1.610±0.08^B^
**G5**	0.028±0.02^A^	0.625±0.07^D^	26.698±1.40^D^	14.971±0.48^C^	1.602±0.03^B^
**G6**	0.000±0.00^A^	0.692±0.12^BD^	32.684±1.08^E^	19.109±0.60^A^	1.659±0.02^AB^

* *Different letters in the same column represent statistical difference, P<0.05*

***Tooth selection and preparation***

The Human Research Ethics Committee of the Health Sciences Institute of the Federal University of Pará (CEP-ICS / UFPA-1.747.518) approved the research protocol for this study. Thesample number was calculated from a pilot test, based on the values for a percentage of Ca concentration. The level of significance was 5%, with an effect of 0.80. The sample size was fixed in five teeth for each group. Thirty extracted single-rooted human teeth were selected according to the following criteria: teeth had mature apices and apical diameters compatible with a size 10 K-file (Dentsply Maillefer, Ballaigues, Switzerland). Teeth with caries, cracks, root canal atresia and calcification were excluded. Remnants of debris and soft tissue on the tooth surfaces were removed, and all teeth were stored in a saline solution at 9^°^ C until use.

The teeth were decoronated at the cementoenamel junction using a low-speed diamond disk (KG Sorensen, São Paulo, SP, Brazil) under constant cooling. The canals were explored with #10 K-files until it was just visible at the apical foramen, and 1 mm from this point was subtracted to standardize the root apical limit and ensure that only the canal dentin was analyzed. 

Then, longitudinal grooves were prepared on the buccolingual surfaces of each root using a diamond disc (KG Sorensen, São Paulo, SP, Brazil) at low speed without penetrating the canal. The roots were then split into two halves using a chisel. The halves were immersed for 10 min in an ultrasonic tub with deionized water for cleaning. Then, the samples were randomized, ensuring that the two halves of the same tooth did not remain in the same group. The samples were randomly divided into 6 groups according to irrigant solutions and irrigation protocols and are presented in Table 1.

The times for each substance were taken as previously described in literature [13]. In all groups, the immersion solution was renewed every 15 min. The substances remained under constant agitation in an ultrasonic tub at 40 kHz/300 W (BioWash Td 30 Plus, BioArt, Equipamentos Odontológicos LTDA, São Paulo, SP, Brazil). The ten halves of each group were divided as follows: five halves were used for SEM and EDS analysis, and five were used for XRD analysis.

***Scanning Electron Microscopy (SEM)***

SEM was used to analyze the ultrastructural changes of dentin. The samples were mounted on metallic stubs with the canal light facing up, gold sputtered and examined under a scanning electron microscope (LEO-1430, Carl Zeiss, Oberkochen, BW, Germany). Several photomicrographs were obtained to observe the surface morphology under 1000× magnification of the canal walls at the coronal, middle, and apical thirds of each specimen.

The level of erosion was evaluated 2 mm near the light of the root canal in each wall of the root thirds. Two examiners performed blind evaluations independently. Kappa test was verified the intra- and inter-examiner reliability. Previous studies in the literature [19, 20] were used to classify the degree of erosion of the dentinal tubules as follows: a score of 0 was indicative of no erosion (all tubules presented a normal appearance and size), a score of 1 was indicative of moderate erosion (the peritubular dentin was eroded). Score of 2 was indicative of severe erosion (the dentin was totally eroded, and the tubules were connected to each other). The control group was excluded because it was impossible to visualize the dentinal tubules [19, 20].

**Table 3:** Comparison by one-way ANOVA (*P*<0.0001) and multiple comparison by the Tukey’s test (*P*<0.05) of the means and standard deviations of the chemical elements detected for the middle third of the teeth in the different experimental groups (*n*=5)

**Groups**	**Chemical Elements of Root Dentin (w%)**				
	**K**	**Mg**	**Ca**	**P**	**Ca/P**
**G1**	0.031±0.01^A^	0.788±0.15^AD^	35.750±2.73^A^	18.975±0.47^A^	1.898±0.18^A^
**G2**	0.041±0.03^A^	0.346±0.12^B^	15.106±1.53^B^	8.803±1.68^B^	1.629±0.02^B^
**G3**	0.043±0.03^A^	0.982±0.08^AC^	32.172±0.86^C^	18.929±0.33^A^	1.678±0.02^B^
**G4**	0.027±0.02^A^	0.973±0.03^AC^	32.654±0.51^C^	18.375±0.40^A^	1.782±0.05^AB^
**G5**	0.013±0.00^A^	0.691±0.04^D^	27.122±0.25^D^	16.252±0.62^C^	1.709±0.01^B^
**G6**	0.074±0.07^A^	0.737±0.09^D^	27.133±0.83^D^	16.597±0.40^C^	1.873±0.06^A^

* *Different letters in the same column represent statistical difference, P <0.05*

**Table 4:** Comparison by one-way ANOVA (*P*<0.0001) and multiple comparison by the Tukey’s test (*P*<0.05) of the means and standard deviations of the chemical elements detected for the apical third of teeth in the different experimental groups (*n*=5).

**Groups**	**Chemical Elements of Root Dentin (w%)**				
	**K**	**Mg**	**Ca**	**P**	**Ca/P**
**G1**	0.062±0.05^A^	0.651±0.05^A^	27.902±3.50^A^	16.806±1.80^A^	1.741±0.02^A^
**G2**	0.026±0.02^A^	0.373±0.07^B^	20.538±1.36^B^	11.774±0.64^B^	1.569±0.04^B^
**G3**	0.043±0.04^A^	0.858±0.06^C^	24.371±2.24^C^	11.773±1.03^C^	1.595±0.05^B^
**G4**	0.028±0.02^A^	1.288±0.12^D^	27.660±1.98^AC^	16.612±0.66^A^	1.610±0.08^B^
**G5**	0.030±0.01^A^	0.614±0.06^A^	26.653±1.14^AC^	16.552±0.40^AC^	1.602±0.03^B^
**G6**	0.021±0.02^A^	0.527±0.14^AB^	22.080±2.26^B^	13.759±1.13^D^	1.659±0.02^AB^

* *Different letters in the same column represent statistical difference, P<0.05.*

**Figure 1:**
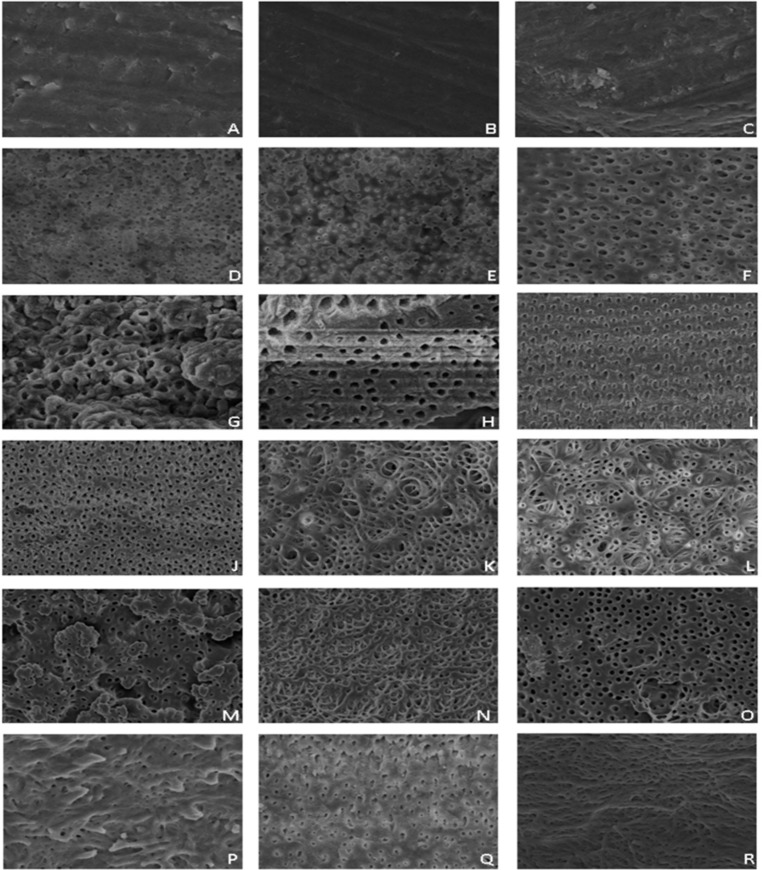
Photomicrographs of human root dentine from the cervical, middle and apical thirds of groups 1 (*A*, *B* and *C*), 2 (*D, E* and *F*) 3 (*G, H* and *I*), 4 (*J, K*, and *L*), 5 (*M, N* and *O*) and 6 (*P, Q* and *R*) (original magnification 1000×, 20 kV)

***Energy Dispersive X-ray Spectrometry (EDS)***

Chemical characterization (wt%) was performed by EDS (LEO-1430; Carl Zeiss, Oberkochen, BW, Germany). The concentrations of the following elements were evaluated: K, Mg, Ca, and P; and the Ca/P ratio on the root third of each group was determined.

The mineral content was not determined before the treatment of each sample because this analysis requires a destructive method. Thus, it is likely that the mineral content differed between all teeth used. However, equal numbers of halves were used for each experimental group in addition to the random process, ensuring that the samples were divided randomly in each group, in addition to the control group, in accordance with other authors who evaluated the chemical composition of root dentin [4, 8, 11].

***X-RAY Diffraction (XRD)***

After the irrigation protocols, the fragments were dried and stored in a biological incubator at 37 ^°^C for 24 h. The analysis of changes in the crystal phases was performed using XRD with a diffractometer (PW 3040/60; X´Pert Pro MPD, PANalytical, São Paulo, SP, Brazil). The following instrumental conditions were used: a 4^°^ to 70^°^ 2θ scan range at 40 kV and 40 mA, with a step of 0.02^°^ in 2θ; a time/step of 20 s; 1/2 slit fixed and one anti-scattering 10 mm mask; and sample movement spinning at 1 rps. Data acquisition was performed using the software X'Pert Data Collector 2.1.

**Figure 2:**
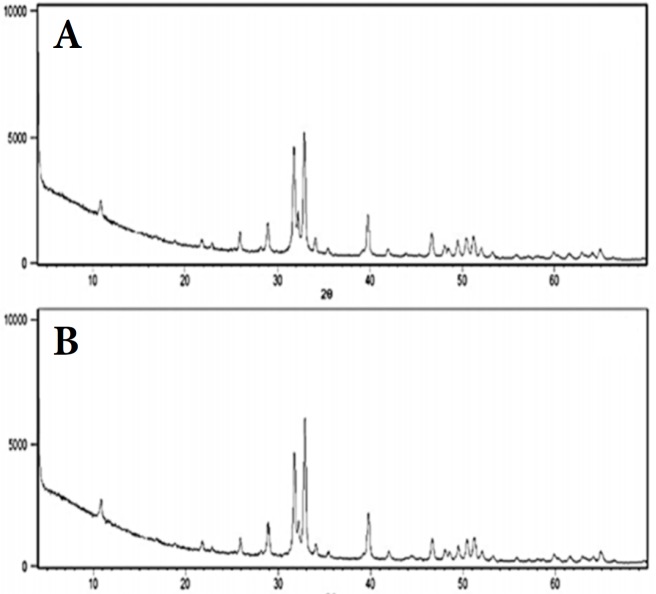
Representative XRD patterns of groups 1 to 4; *A)* hydroxyapatite peak corresponding to 5 and 6 and; *B)* the peak corresponding to fluorapatite

***Statistical analysis***

A descriptive analysis was performed for the data in the ultrastructure and crystallography of dentin. Analysis of variance (one-way ANOVA) followed by multiple comparison tests (Tukey’s tests) were performed by IBM® SPSS® Statistics 20 software (SPSS, Chicago, IL, USA) on the chemical composition data of dentin. The mean and standard deviation of the data from the cervical, middle and apical thirds were determined. All hypothesis testing was performed at a 95% confidence level.

## Results

***SEM***

The images shown in Figure 1 represent the most common aspect observed in the different thirds of the samples. G1 predominantly showed the presence of the smear layer covering the dentin wall, obliterating the dentinal tubules (Figure 1A to C).

In G2, the cervical and middle thirds had exposed dentinal tubules with preserved intertubular dentin. In the apical third, there were several areas of erosion of intertubular dentin and irregularly enlarged tubules, and, in some areas, excessive erosion led to the communication of dentinal tubules (Figure 1D to F). In G3, there was irregular intertubular dentin with an uneven appearance and some debris near the entrances of the tubules (Figure 1G to I).

In the cervical third of G4, the presence of micro branches was noted. The entry of the tubules was irregular. In the middle and apical thirds, there was a greater opening of the tubules and erosion areas, leading to the union of two or more tubules. There was a reduction in the space occupied by the intertubular dentin (Figure 1J to L). In G5, the intertubular dentin was free of debris, and the tubules were clearly observed (Figure 1M to O). G6 showed the presence of a smear layer in the dentinal walls, particularly in the apical third, covering part of dentinal tubules. The intertubular dentin showed an irregular surface (Figure 1P to R).

The obtained intra-examiner Kappa was 0.972 for erosion analysis, and 0.813 was obtained for the inter-examiner analysis. There was no statistically significant difference between the degree of erosion of samples from groups G2 to G6 (*P*=0.554).

***EDS***

Tables 2 to 4 show the K, Mg, Ca, P and Ca/P values, which are expressed in percentages relative to the total amount of all detectable elements in the areas examined in the cervical, middle and apical thirds of each group after treatment with irrigating solutions.

***XDR***

The diffractogram of groups 1 to 4 showed peak characteristics of hydroxyapatite crystals, while those of groups 5 and 6 showed fluorapatite. For the other peaks, no difference was observed when compared to the control group.

## Discussion

Irrigation is an effective method for cleaning the root canal [8]. However, it has been reported that some chemical agents cause changes in the structure of dentin [1, 21]. About irrigator solutions, there is no consensus regarding the contact time with the dentin surface, and a period of less than 1 and up to 15 min has been reported . About irrigator solutions, there is no consensus regarding the contact time with the dentin surface, and a period of less than 1 and up to 15 min has been reported [4, 7, 22]. For the present study, 3 min was adopted [6]. Regarding HEBP, both for single use and single substances, times and proportions were considered based on previous studies [14, 15].

Flame photometry, SEM, EDS and secondary ion mass spectrometry are recommended techniques for the analysis of the microstructure and measurement of mineral content levels of dentin [1, 3, 23]. In the present study, the ultrastructure of dentin was analyzed using SEM techniques, chemical composition and Ca/P ratio through EDS and crystalline conformation by XRD. The results showed that the sequence and type of irrigation used have an impact on the structure of dentin in the different thirds. In G2 and G4, the presence of dentinal erosion was detected and dentinal tubules were enlarged. Dentin has a dense collagen network covered by hydroxyapatite [3, 4]. NaOCl is a strong base and a non-specific oxidant [8]. When used before EDTA, the hydroxyapatite coating appears to protect the collagen fibers, avoiding direct NaOCl action. However, when used after EDTA, NaOCl acts directly on the widely exposed collagen by the demineralization of the chelating agent [4, 8, 24]. In both groups, it is believed that the contact of the NaOCl with the organic part of dentin may have caused these changes.

Mirseifinejad *et al. *[24] also observed the effects caused in the collagen matrix under irrigation with 17% EDTA followed by 5.25% NaOCl irrigation. The activity of 17% EDTA leads to the demineralization of dentin and, consequently, the exposure of the collagen matrix, which is then dissolved by the 5.25% NaOCl. These findings showed the action of these two irrigating solutions on the organic and inorganic components of the amorphous particles of the smear layer removed [24].

Regarding the loss of mineral structure related to the use of NaOCl, the apatite crystals found in dentin tissue do not fully protect the collagen layer from chemical oxidation. This reaction promotes the loss of proteins from the organic components of the dentin. The acidification process promoted by the OCl^-^ anion contributes also to the loss of mineral structure of the dentin when irrigated by NaOCl, despite the alkaline nature of this solution [25-27]. A “ghost mineral layer” of sparse collagens is formed by irrigation with NaOCl, generating a friable mineral matrix resulting in reduction of flexural strength as a deleterious effect, which may reflect the susceptibility to root fracture. The extent of negative effects on dentin increases with the use of higher concentrations of NaOCl, suggesting that the removal of the organic phase of the mineralized dentine occurs by diffusion with a relation of time and concentration dependence [25-27].

The structural change can also be associated with the amount of mineral content of dentin and the depth of penetration of irrigant. The main inorganic components of the dentin are Ca and P, which are present in the hydroxyapatite crystals [3]. Due to its chelating action, EDTA can cause changes in dentin by modifying the Ca/P ratio [23, 28], including changes in hardness, permeability and solubility properties [4, 28, 29]. G2 had lower Ca and P values in all root thirds and changes in the Ca/P ratio in the apical third. This greater decalcification, evidenced by mineral loss, may have provided the greatest contact of NaOCl with the dentin surface, which may have caused changes such as turning a collagen-rich tissue in an irregular structure and increasing dentinal tubules and erosion areas in G2.

Regarding G4, it was believed that the combined use of 5% NaOCl and 18% HEBP promoted the direct action on collagen fibers, resulting in greater tubular opening. Decreases in Ca, P and the Ca/P ratio were not significant in the apical third. It has been suggested that the HEBP may have led to a more superficial action of NaOCl on the organic portion of the dentin, as EDTA has a greater action on inorganic components [8]. EDTA removed effectively the smear layer by chelating action, however due to erosive effect; causes changes in the mechanical properties of dentin [30].

The results showed higher levels of Ca in the groups treated with HEBP. In relation to P, the mineral pattern was similar. These results may be closely associated with the chelating ability of the solutions used. Regarding the structural changes in G3, because a weak chelator was used (HEBP), the action of the final rinse with NaOCl was limited to the smear layer, and it probably did not reach the underlying dentin. In the final irrigation, samples of G5 and G6 were immersed in a solution that had no action on organic matter, which might have prevented the occurrence of dentin erosion. In addition to the presence of Ca and P, small amounts of Mg were detected in mineralized tissues. The Mg appears to influence the mineralization process. The absence of this element reduces the number of odontoblasts, inhibiting and/or reducing the formation of dentin [11]. NaOCl is able to remove Mg ions [28]. The Mg values in each third of the root canal are in agreement with the study of Cobankara *et al.* although the apical third of G4 showed the highest values [4]. 

There are limited information about the role of K, which is present within the cells [4]. In this study, there was no significant statistical difference in the reduction of K among all groups. Despite the mineral loss, no changes were observed in the crystalline conformation of dentin, with no formation of by-products capable of altering or forming new compounds.

Thus, the null hypothesis, which stated that there would be no change in the morphology, chemical components and crystals of dentin after contact with different types of irrigation solutions and protocols, was rejected. However, it was accepted for the crystalline conformation.

## Conclusion

The results obtained in the experimental conditions of this study showed that the combined use of irrigating solutions with direct action on the organic and inorganic matter promoted the most significant changes compared to the other groups. It was observed that the level of these changes was determined by the chelation ability of the demineralizing agent. However, there were no significant differences observed crystallographically.
